# Multilocus Sequence Typing and Antifungal Susceptibility of *Candida albicans* Isolates From Milk and Genital Tract of Dromedary Camel

**DOI:** 10.3389/fvets.2022.905962

**Published:** 2022-07-08

**Authors:** Mahmoud M. Fayez, Ayman A. Swelum, Nada K. Alharbi, Ahlam H. AlRokban, Abdullah Almubarak, Ameen H. Almubarak, Fanan Alaql, Ahmed E. Ahmed

**Affiliations:** ^1^Department of Bacteriology, Veterinary Serum and Vaccine Research Institute, Ministry of Agriculture, Cairo, Egypt; ^2^Al Ahsa Laboratory, Ministry of Agriculture Kingdom of Saudi Arabia, Al Ahsa, Saudi Arabia; ^3^Department of Theriogenology, Faculty of Veterinary Medicine, Zagazig University, Zagazig, Egypt; ^4^Department of Biology, College of Science, Princess Nourah bint Abdulrahman University, Riyadh, Saudi Arabia; ^5^Riyadh Veterinary Diagnostic Lab, Ministry of Environment, Water and Agriculture, Riyadh, Saudi Arabia; ^6^Biology Department, College of Science, King Khalid University, Abha, Saudi Arabia; ^7^Department of Theriogenology, Faculty of Veterinary Medicine, South Valley University, Qena, Egypt

**Keywords:** MLS, Candida, camel, fertility, infertility

## Abstract

Multilocus sequence typing (MLST) was used to study the genetic diversity and population structure of 48 *Candida albicans* (*C. albicans*) isolates from the udder or genital tract of apparently healthy or diseased camels. This study aimed also to determine the frequency of *C. albicans* isolates in the genital tract and udder of healthy or diseased female dromedary camels. A total of 240 mature dromedary camels (230 females and 10 males) were categorized based on the clinical examination of gentile tract and udder into five groups [fertile females (*n* = 70), infertile females (*n* = 115), healthy udder (*n* = 15), mastitis (*n* = 30), and fertile males (*n* = 10)]. Swabs were collected from male and female genital tracts of dromedary camels and milk samples were collected from healthy and diseased udders. *C. albicans* was isolated from 20% of the samples. The frequency of isolation was significantly higher (*p* < 0.00001) in disease camels (75%) compared with apparently healthy camels (25%). Most of *C. albicans* was isolated from infertile female genitalia (62.50%) which was significantly higher than that isolated from fertile female genitalia (16.67%). Multilocus sequence (MLS) analysis identified seven different diploid sequence types (DSTs) including DST2, DST50, DST62, DST69, DST124, DST142, and DST144. The most frequently identified DTS was DST69 (13/48) which significantly higher (*p* ≤ 0.05) than DST2, DST62, and DST124. The frequency of identification of DST50, DST142, and DST 144 was significantly higher (*p* ≤ 0.05) than DST62. DST62 and DST124 were isolated only from diseased camels. DST62 was isolated only from mastitic milk. DST124 was isolated only from infertile female genitalia. The percentage of DST50 and DST 142 was significantly higher in diseased camels (infertile females) than in the apparently healthy ones (fertile females). DST2 and DST50 were isolated only from female genitalia of apparent health and diseased camels. The *C. albicans* isolated from diseased camels had significantly higher biofilm formation, hydrophobicity, phospholipase, proteinase, and hemolysin activities compared with the isolates from apparent healthy camels. All isolates were sensitive to amphotericin B, itraconazole, micafungin, posaconazole and voriconazole. In conclusion, the present study represents the first molecular typing of *C. albicans* in samples isolated from milk and the genital tract of the dromedary camel. MLST is a useful tool for studying the epidemiology and evolution of *C. albicans*. Early identification of *Candida* species and attention to *Candida* virulence factors and their antifungal susceptibility patterns is very important for establishing strategies to control and/or prevent candidiasis by novel therapeutic management. Amphotericin B, itraconazole, micafungin, posaconazole, or voriconazole can be efficient in treatment of candidiasis.

## Introduction

Microbial organisms are responsible for many diseases that directly or indirectly affect reproductive success in Camelidae (1) Genital tract inflammation (GTI), caused by microbial infections, is the main cause of infertility in different animal species including camels (2). Mastitis and GTI reduce milk yield, productivity, and profitability of dairy farms. This reduction in production performance is associated with substantial economic losses on dairy farms. Microbial infection, bacterial, fungal, or viral, is the main cause of mastitis and GTI. Local fungal infections, especially in the animal, can be overlooked in camel care and treatment. The incorrect treatment protocols, which depend mainly on using of antibiotics and hormonal therapy, can lead to systemic fungal infections and adverse outcomes.

Many fungal agents including different Candida species are found in the mucous membranes of various animals and humans including *C. albicans, C. tropicalis, C. parapsilosis, C. glabrata, C. krusei, C. aloffi, C. bovina*, and *C. keyfr*. *Candida* sp. Is the most common fungal agent in raw camel milk (3–5) *C. albicans* is isolated from the apparent healthy or inflamed genital tract of cows and buffalos (6); horses (7, 8), dogs (9) and camels (10). Some of *Candida* species, especially *C. albicans*, can cause lesions that are mostly localized in the gastrointestinal tract and occasionally in the skin, subcutaneous tissues, lung, uterus, breast, testis, and other organs (11). *C. albicans* grabs the opportunity of any predisposing factors, causing debilitation or immunocompromising, and become a systemic pathogenic infection by spreading to different organs through the blood. Systemic candidiasis is a very serious infection that can affect the blood, heart, brain, eyes, bones, and other parts of the body and cause fever, murmurs sound in the heart, oliguria, blindness, and/or splenomegaly (12–14).

Several genotyping methods have been used to study the molecular epidemiology of *C. albicans*, including pulsed-field gel electrophoresis (PFGE), restriction fragment length polymorphism (RFLP), and random amplified fragment length polymorphism (RAPD); these methods are labor-intensive, time-consuming and their results are difficult to compare among laboratories (15). However, multilocus sequence typing (MLST), as a relatively new tool based on DNA sequencing, exhibits high discriminatory power and reproducibility, which overcome the flaws of more subjective methods, making it possible to compare results among laboratories (16). Recently, a standard MLST protocol for molecular characterization of *C. albicans* has been proposed based on the sequences of seven housekeeping genes (AAT1a, ACC1, ADP1, MPI1b, SYA1, VPS13, and ZWF1b) (17). This method has been widely used to study the population structure, transmission, and microevolution of *C. albicans*.

Knowledge of persistent and transient microflora living in the animals' genital tract and udder is important for a better understanding of the pathological processes and subsequently proper treatment. Molecular epidemiology that combines traditional epidemiological investigation with molecular typing is useful for identifying community or nosocomial infections and tracing the source of transmission and outbreaks. To our knowledge, little data on *C. albicans* of female genitalia and udder and their impact on GTI and mastitis have been described in dromedary camels. Because of the potential role of *C. albicans* as endogenous sources of infections, identifying the *C. albicans* in the normal and pathological genital tract and udder will contribute to our understanding of their pathological role. The purposes of this study were to provide current data on *C. albicans* and to determine the frequency of *C. albicans* isolates in the genital tract and udder of healthy or diseased female dromedary camels.

## Materials and Methods

### Animals

A total of 240 mature dromedary camels (230 females and 10 males) were included in the current study. All these camels were located in the eastern region, of the Kingdom of Saudi Arabia. A total of 145 of these animals (39.58%) were apparently healthy. While 60.42% of them (*n* = 95) were diseased animals. These camels were categorized based on the clinical examination of the gentile tract and udder into five groups. The fertile females group (*n* = 70) had a history of normal fertility and apparent healthy under clinical examination. The infertile females group (*n* = 115) had a history of infertility and clinical examination revealed GTIGTI. The healthy udder group (*n* = 15) had apparent healthy udder and normal milk. The mastitis group (*n* = 30) suffered from clinical mastitis. The fertile males group (*n* = 10) had a history of normal fertility and were apparently healthy.

### Samples

A total of 195 swabs were collected from the male (*n* = 10) and female (*n* = 185) genital tracts of dromedary camels after proper cleaning of the external genitalia. Briefly, the tail of the female camel was wrapped and held out of the way. The perineal area or udders of females and ventral abdomen of males were washed with a non-residual soap and thoroughly rinsed with clean water. The region was dried with disposable paper towels. A small moist piece of paper towel was used to gently wipe the inside of the vestibule. The guarded swab (minitube, Germany), which consisted of an introduction pipette and pre-perforated cap, was used for sampling without risk of contamination. Swabs were inserted directly into AMIES transport medium to avoid dehydration of the samples. Milk samples were collected from healthy (*n* = 15) and diseased (*n* = 30) udders in sterile containers following the standard methods described by Hogan et al. (18). All samples were collected following the Local Ethics Committee for animal research in the Kingdom of Saudi Arabia. Samples were labeled and sent cooled to the laboratory for mycological investigation.

### Isolation and Phenotypic Identification of *Candida* Species

Milk samples and swabs were cultivated on to Sabouraud dextrose agar (SDA) (Oxoid, England) supplemented with chloramphenicol (100 mg/L) and CHROMagar *Candida* (CHROMagar™, Paris, Francs) and incubated aerobically at 37°C for 24–48 h. Yeast colonies were visually inspected and 3–5 green colonies were purified on SDA. Isolated were biochemically identified by VITEK^®^ 2 compact using VITEK^®^ 2 YST ID card (bioMérieux, Francs).

### Molecular Identification of Isolates

The genomic DNA was purified using QIAamp^®^ DNA Mini Kit (QIAGEN, Francs) according to the manufacturer's instructions. The universal primers ITS1 (5′-TCCGTAGGTGAACCTGCGG-3′) and ITS4 (5′-TCCTCCGCTTATTGATATGC-3′) were used to amplify the 5.8S RNA gene and the internal transcribed spacer (ITS) region (19). PCR products were purified (QIAquick PCR Purification Kit, QIAGEN GmbH—Germany) and subsequently sequenced (3500 Genetic Analyzer, Applied Biosystems. CA, USA). All ITS sequences were applied to the BLAST program of National Center for Biotechnology Information (NCBI) Web site (http://blast.ncbi.nlm.nih.gov/) to analyze and determine their actual species. Alignment and phylogenetic reconstructions were performed using the function “build” of ETE3 v3.1.1 (20) as implemented on the GenomeNet (https://www.genome.jp/tools/ete/). The tree was constructed using the Interactive Tree of Life (iTOL) v6.4.3 tool (https://itol.embl.de/) (21).

### Multilocus Sequence Typing of *C. albicans* Isolates

MLST was performed based on the sequencing of seven housekeeping genes (AAT1a, ACC1, ADP1, MPIb, SYA1, VPS13, and ZWF1b) (17). Sequences from the seven loci were assigned to genotypes and the diploid sequence types (DSTs) were obtained by comparing the sequence with the reference *C. albicans* MLST database (https://pubmlst.org/calbicans/).

### Biofilm Formation Assay

The ability of *C. albicans* isolates to form biofilm was evaluated (22). *C. albicans* isolates were harvested from SDA in Sabouraud dextrose broth (SDB) (Oxoid, England) and adjusted spectrometrically to be equivalent to 3 × 10^7^ CFU/ml; then after 20 μl was transferred to sterile 96 well microtiter plate (Nunc™, MicroWell™, Denmark) containing 180 μl of SDB supplemented with 8% glucose. All plates were sealed and incubated for 24 h at 35°C with gentle shaking. The plates were washed 3 times with sterile distilled water to remove the planktonic cells. After adding 200 μl of sterile distilled water to each well, biofilm formation was directly measured by spectrophotometric reading method at 405 nm (Elx808 Absorbance Reader, BioTek, Germany). The absorbance readings were converted to percent transmittance (%T). The %T value for each test sample was subtracted from the %T value for the reagent blank to obtain a measure of the amount of light blocked when passing through the wells (%Tbloc). Biofilm production by each isolate was scored as either negative (%Tbloc, <5), 1+ (%Tbloc, 5–20), 2+ (%Tbloc, 20–35), 3+ (%Tbloc, 35–50), or 4+ (%Tbloc, ≥50) (22). Based on this score, biofilm producing *C. albicans* were further classified as weak (1+), moderate (2+, 3+), and strong (4+) positive.

Biofilm was quantified biochemically using the XTT reduction assay (XTT, Sigma) (23) and the XTT assay absorbance was measured spectrophotometrically at 490 nm (Elx808 Absorbance Reader, BioTek, Germany) and the percentage transmittance (%T) was calculated. Biofilm production was scored as 1+ (%T >60), 2+ (%T 41–60), 3+ (%T 21–40), 4+ (%T 11–20), 5+ (%T 6–10), or 6+ (%T ≤ 5). Each isolate was examined in triplicate on two different days and the reference strain *C. albicans* ATCC 90028 was used as study control in both assays.

### Determination of Proteinase Activity

The proteinase activity of *C. albicans* isolates was assayed on solid media (24). An Aliquot of 10 μl from overnight growth yeast suspension containing (1 × 10^6^ CFU/ml) was spread over bovine serum albumin agar (BSAA). The plates were incubated for 7 days at 28 oC and the proteolysis of BSA was visualized after staining with amido black. The proteinase activity zone (Pz) was calculated as described by Price et al. (25) and scored as 4+ (Pz <0.69), 3+ (Pz 0.79–0.70), 2+ (Pz 0.89–0.80) and 1+ (Pz 0.9–1). Each isolate was examined in triplicate on two different days and the reference strain *C. albicans* ATCC 90028 was used as the study control.

### Determination of Phospholipase Activity

The extracellular phospholipase activity of *C. albicans* isolates was evaluated by the egg yolk agar plate method (26). The plates were inoculated with 10 μl from overnight growth yeast suspension containing (1 × 10^6^ CFU/ml) and incubated at 37°C for 7 days. The zone of phospholipase (PZ) was calculated as described by Price et al. (25) and subsequently isolates were classified into four groups; 1+ (Pz 0.9–1), 2+ (Pz 0.89–0.080), 3+ (Pz 0.79–0.70) and 4+ (Pz <0.69); accordingly, a lower Pz ratio corresponds with higher enzyme activity. Each isolate was examined in triplicate on two different days and the reference strain *C. albicans* ATCC 90028 was used as the study control.

### Determination of Hemolysin Activity

Hemolysin activity was evaluated using the blood plate assay (27). Media were prepared by adding 7 ml fresh sheep blood to 100 ml SDA (Oxoid, England) supplemented with glucose at a final concentration of 3% (w/v). The final pH (mean ± SD) of the medium was 5.6 ± 0.2. A standard inoculum of both the test and the control *Candida* isolates [10 μl, with 108 *candida* cells (ml saline)-1] was deposited onto the medium. The plate was then incubated at 37°C in 5% CO_2_ for 48 h. After incubation, the plates were examined and the ratio of the diameter of the colony to that of the translucent zone of hemolysis (mm) was used as the hemolytic index (Hz value) to represent the extent of hemolytic activity by different *Candida* isolates. The hemolytic index (Hz) was calculated as described by Price et al. (25) and subsequently isolates were classified into four groups; 1+ (Hz 0.9–1), 2+ (Hz 0.89–0.080), 3+ (Hz 0.79–0.70), and 4+ (Hz <0.69). The reference strain *C. albicans* ATCC 90028 was used as a study control.

### Cell Surface Hydrophobicity (CSH) Assay

The microbial adhesion to hydrocarbons (MATH) assay previously described by Rosenberg (28) was used to determine the cell surface hydrophobicity of C. *albicans* isolates. A loop full from overnight growth on SDA at 37°C was suspended and washed twice in phosphate buffer saline (PBS) and adjusted to display OD_600nm_ between 0.4 and 0.5 (A_o_); then 3.0 ml adjusted suspension from each isolate was overlaid with 4.0 ml of the hydrophobic hydrocarbon, n-hexadecane (Sigma Aldrich, St. Louis, MO, USA) and vortexed vigorously for 30 s and left for 10 min at 30°C to allow phases separation. The lower aqueous phase was carefully separated using a glass Pasteur pipette and transferred to a clean polystyrene tube and its OD_600nm_ was measured (A_1_). Results were reported as an average of three independent measurements, which were calculated according to the formula hydrophobicity (%) = [1–(A1/A0)] × 100. All assays are representative of at least three independent experiments, performed in triplicate, and the reference strain *C. albicans* ATCC 90028 was used as the study control.

### Antifungal Susceptibility Testing

Seven different antifungal agents (Fluconazole, Amphotericin B, Flucytosine, Itraconazole Micafungin, Posaconazole, and Voriconazole) were selected for *C. albicans* antifungal susceptibility testing using the broth microdilution test according to the recommendation of the Clinical Laboratory Standards Institute (29). The MIC breakpoints were interpreted (30, 31).

### Statistical Analysis

Comparisons among groups were evaluated using Chi Square (χ^2^) test in all measured parameters except reduction assay and phospholipase, proteinase, and hemolysin activities, which were analyzed by analysis of variance (ANOVA), using SAS (SAS Institute, Cary, NC, USA, 2000). A difference was considered significant at *p*< 0.05 level. Data were expressed in percentages except for reduction assay and phospholipase, proteinase, and hemolysin activities that expressed as the mean ± standard deviation.

## Results

### Isolation and Phenotypic Identification of *Candida* Species

**Figure 1:**
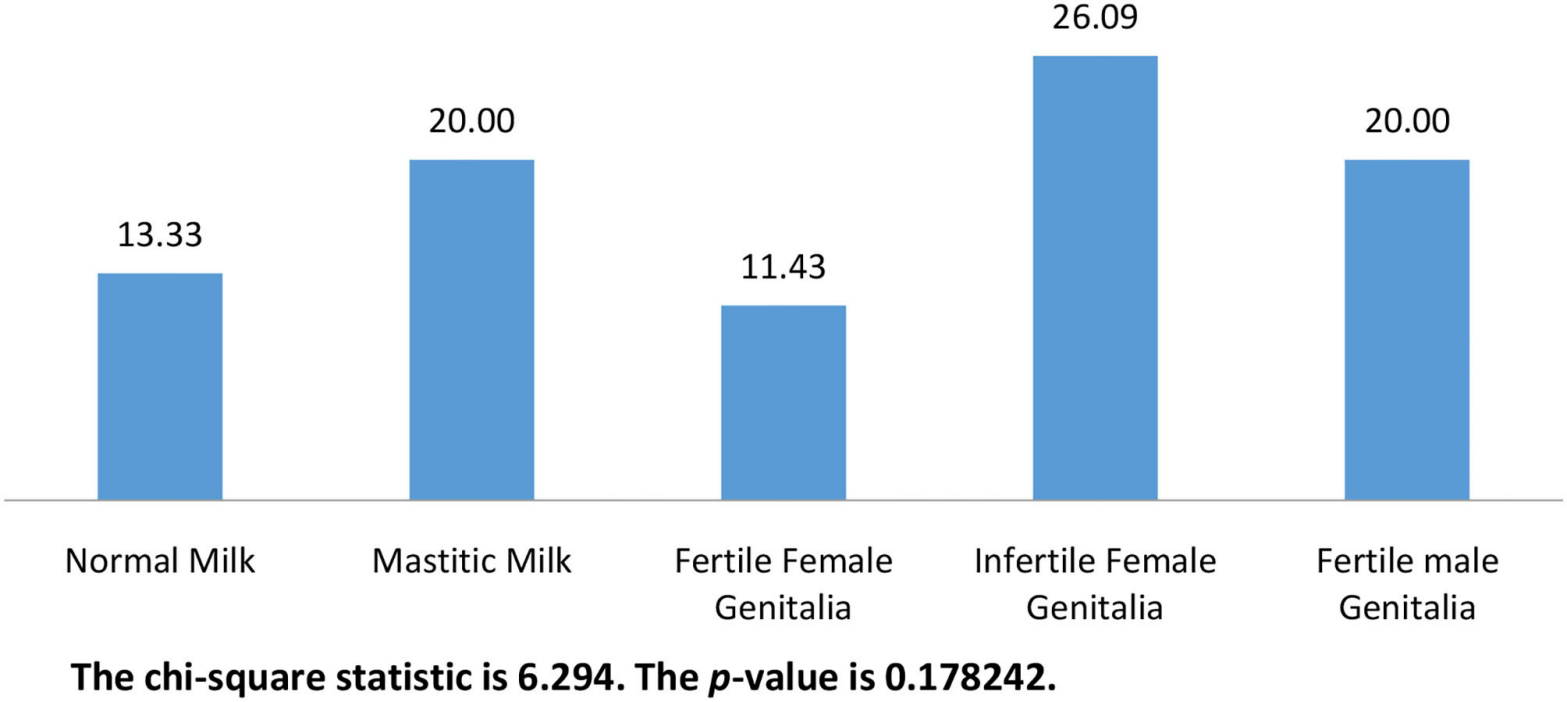
The frequency of isolation of *C. albicans* from different animal groups (*n* = 240).

Overall, 48 (20%) *candida* were isolated out of the 240 samples in this work. The isolates were permissively identified as *C. albicans* based on characteristic growth on chromogenic agar and biochemical profile obtained by the automated VITEK^®^ 2 compact. The frequency of isolation was significantly higher (*p* < 0.00001) in disease camels (75%) compared with apparently healthy camels (25%). *C. albicans* were isolated from 24.83% of diseased females which was significantly (*p* = 0.01682) higher than that isolated from apparently healthy females. Figure 1 shows the frequency of isolation from different animal groups. The percentages of *C. albicans* ranged from 11.43 to 26.09 % without any significant difference (*p* = 0.178242) between different animal groups.

**Figure 2:**
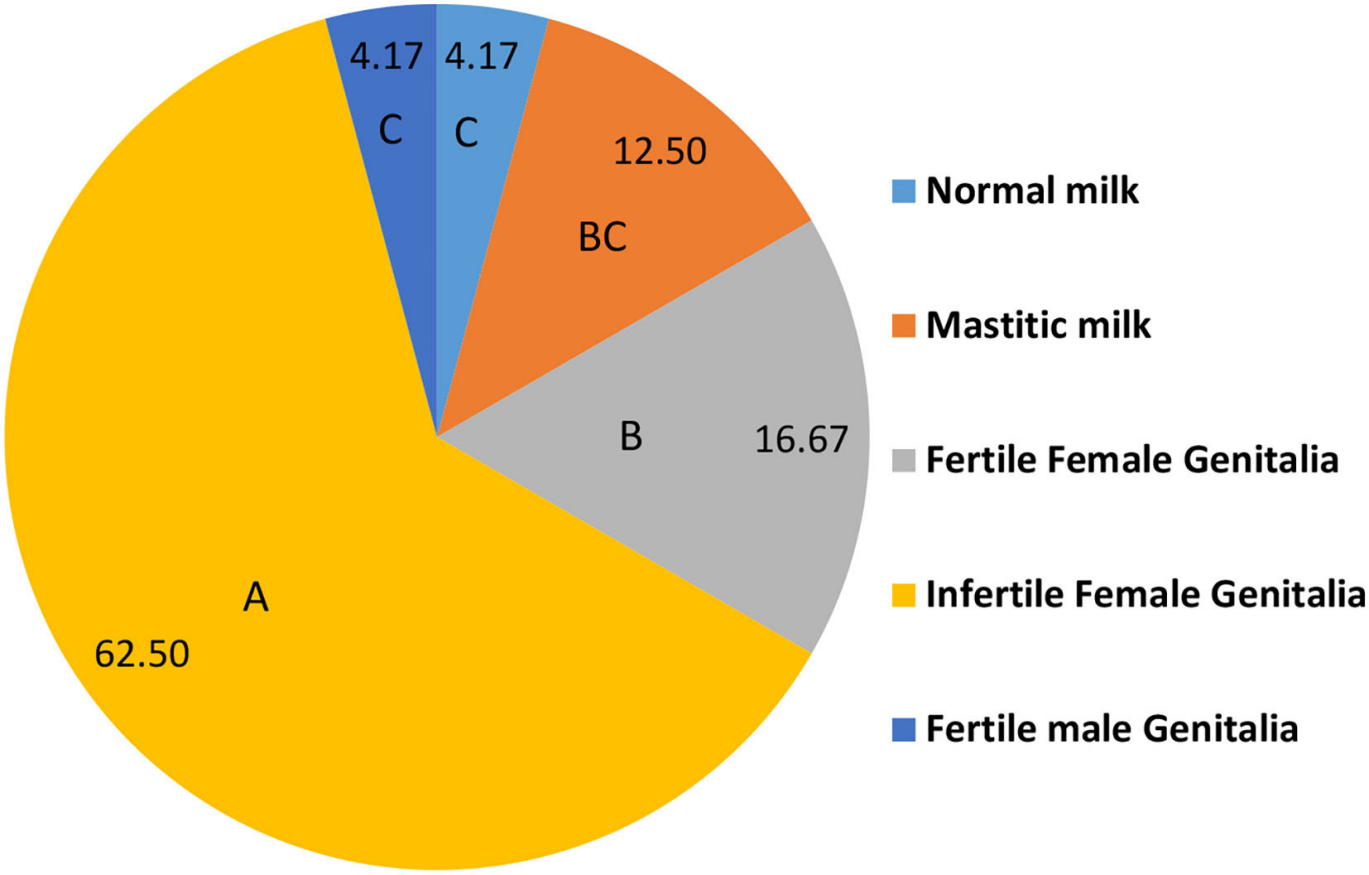
The distribution of isolated *C. albicans* in different positive samples (*n* = 48). A.B.C Percentages carrying different capital letters differed significantly (*p* ≤ 0.05).

Figure 2 shows the distribution of isolated *C. albicans* (*n* = 48) in different samples. Most of *C. albicans* was isolated from infertile female genitalia (62.50%) which is significantly higher than that isolated from fertile female genitalia (16.67%). While, the significantly lowest percentages of *C. albicans* were isolated from normal milk and fertile male genitalia (4.17 and 4.17%, respectively).

### Molecular Identification of Isolates

**Figure 3:**
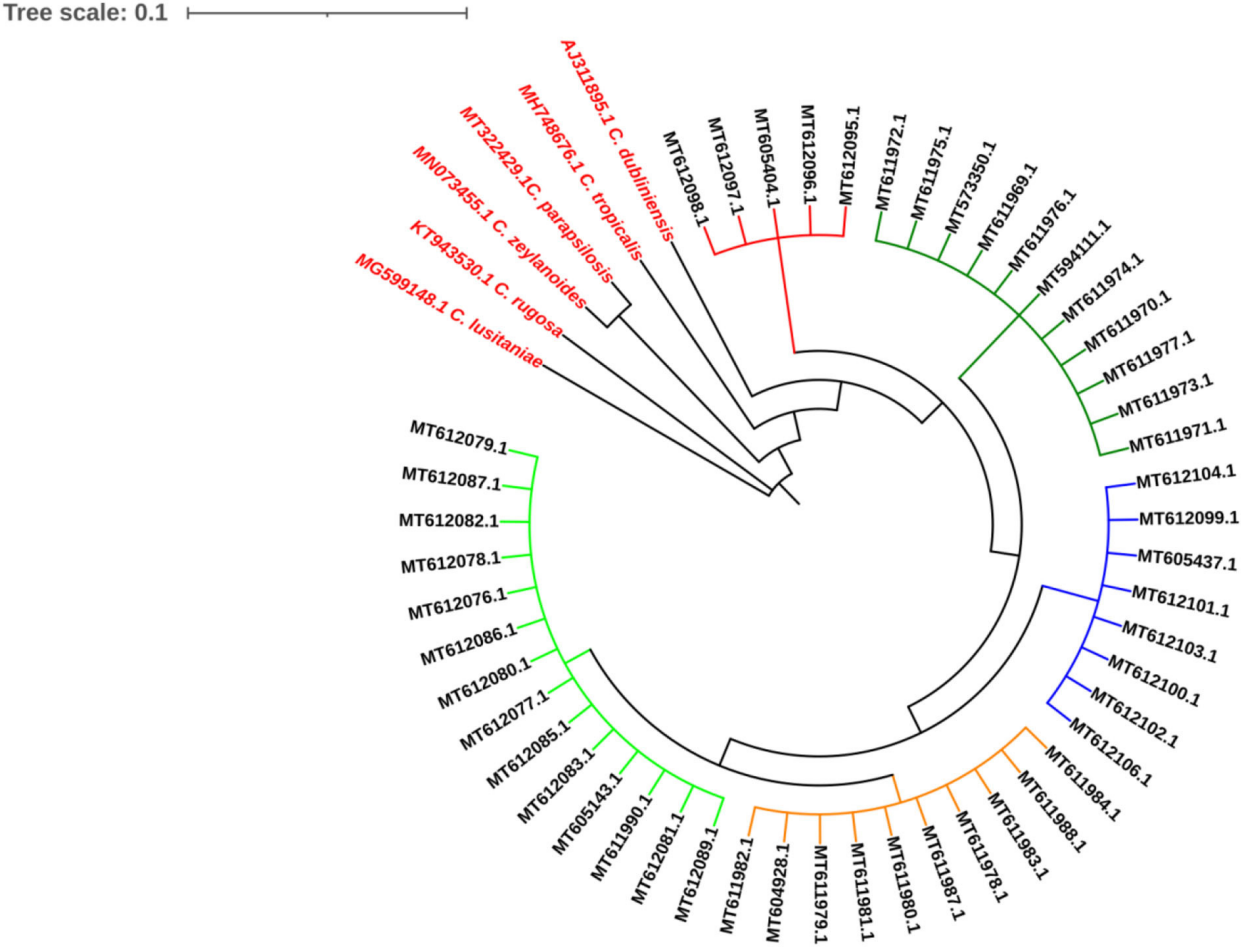
The isolates were genetically identified as *Candida albicans* and clustered in five clades as shown in figure.

The ITS1-5.8SrRNA–ITS2 region from all isolates was amplified and sequenced. All sequences showed a high similarity (>99%) to *C. albicans* database. The sequences were deposited in GenBank under accession numbers shown in Figure 2. Based on ITS1-5.8SrRNA–ITS2 sequence, the isolates were genetically identified as *C. albicans* and clustered in five clades as shown in Figure 3.

### Multilocus Sequence Typing of *C. albicans* Isolates

**Figure 4:**
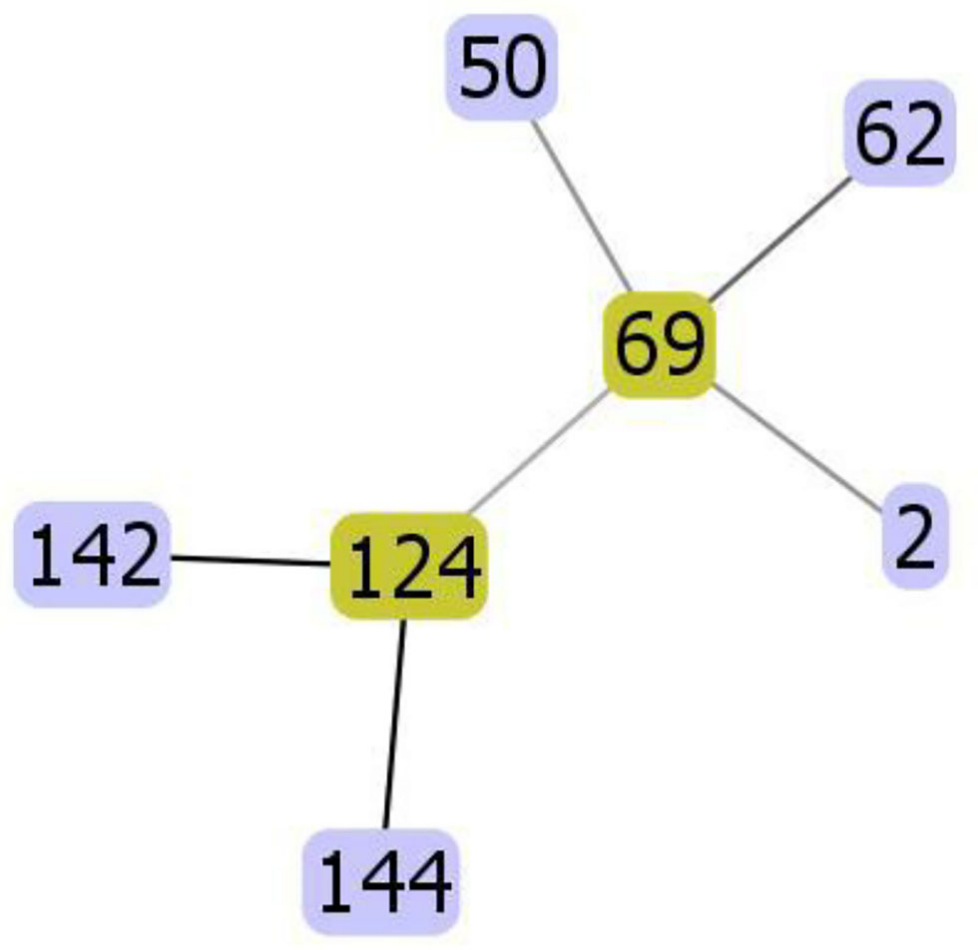
The evolutionary relationships between the diploid sequence types (DSTs) of *C. albicans*. The isolates clustered in two clades; clade 1 (DST2, DST50, DST62, DST69) and clade 4 (DST124, DST142, DST144).

MLS analysis identified seven different DSTs including DST2, DST50, DST62, DST69, DST124, DST142, and DST144. Figure 4 shows the evolutionary relationships between the DSTs. The isolates clustered in two clades; clade 1 (DST2, DST50, DST62, DST69) and clade 4 (DST124, DST142, DST144).

**Figure 5:**
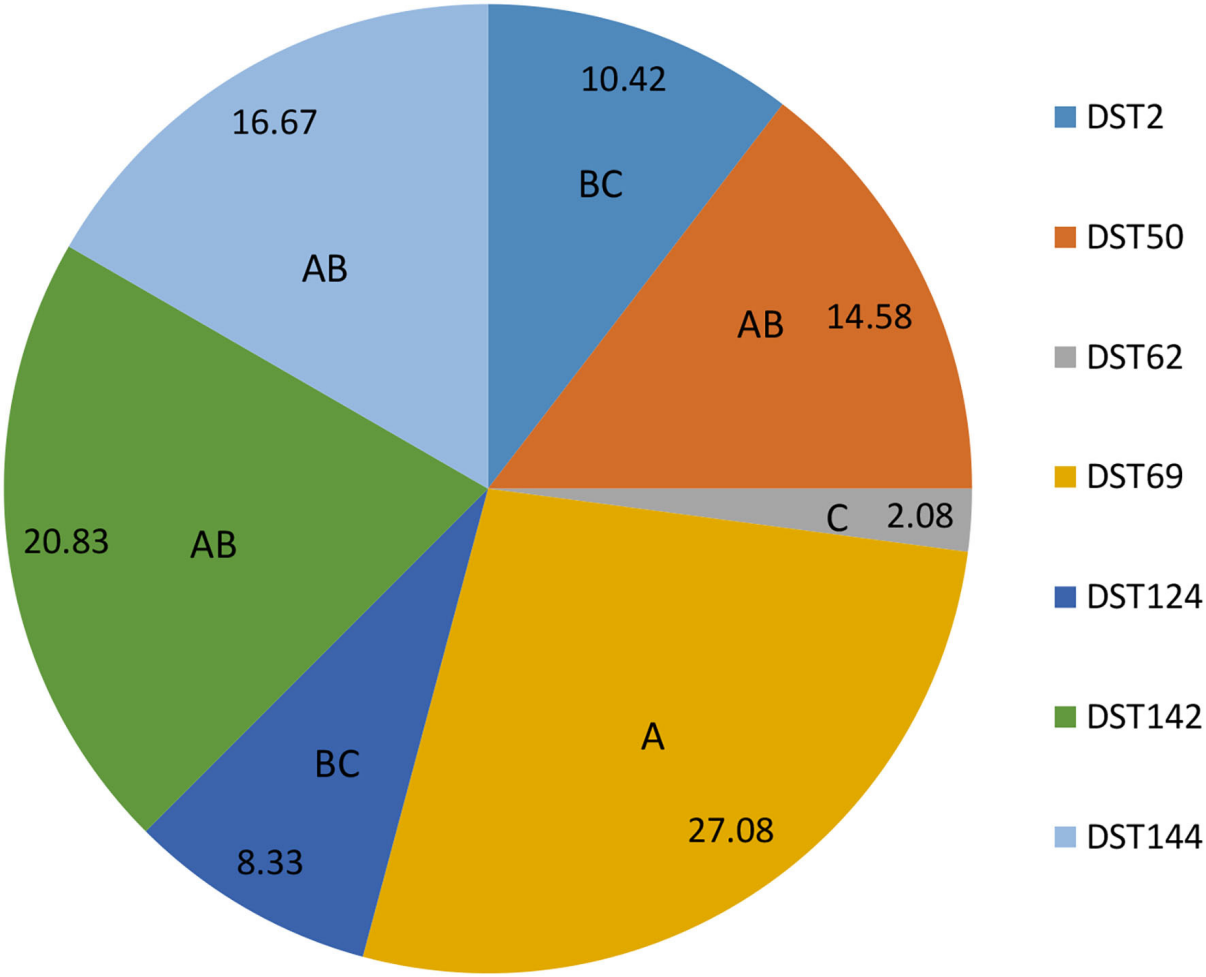
The frequent identification of different diploid sequence types (DSTs) of *C. albicans* isolates from milk and genital tract of dromedary camel. A.B.C Percentages carrying different capital letters differed significantly (*p* ≤ 0.05).

The most frequently identified DTS was DST69 (13/48) which significantly higher (*p* ≤ 0.05) than DST2, DST62, and DST124. The frequency of identification of DST50, DST142, and DST 144 was significantly higher (*p* ≤ 0.05) than DST62 (Figure 5).

**Figure 6:**
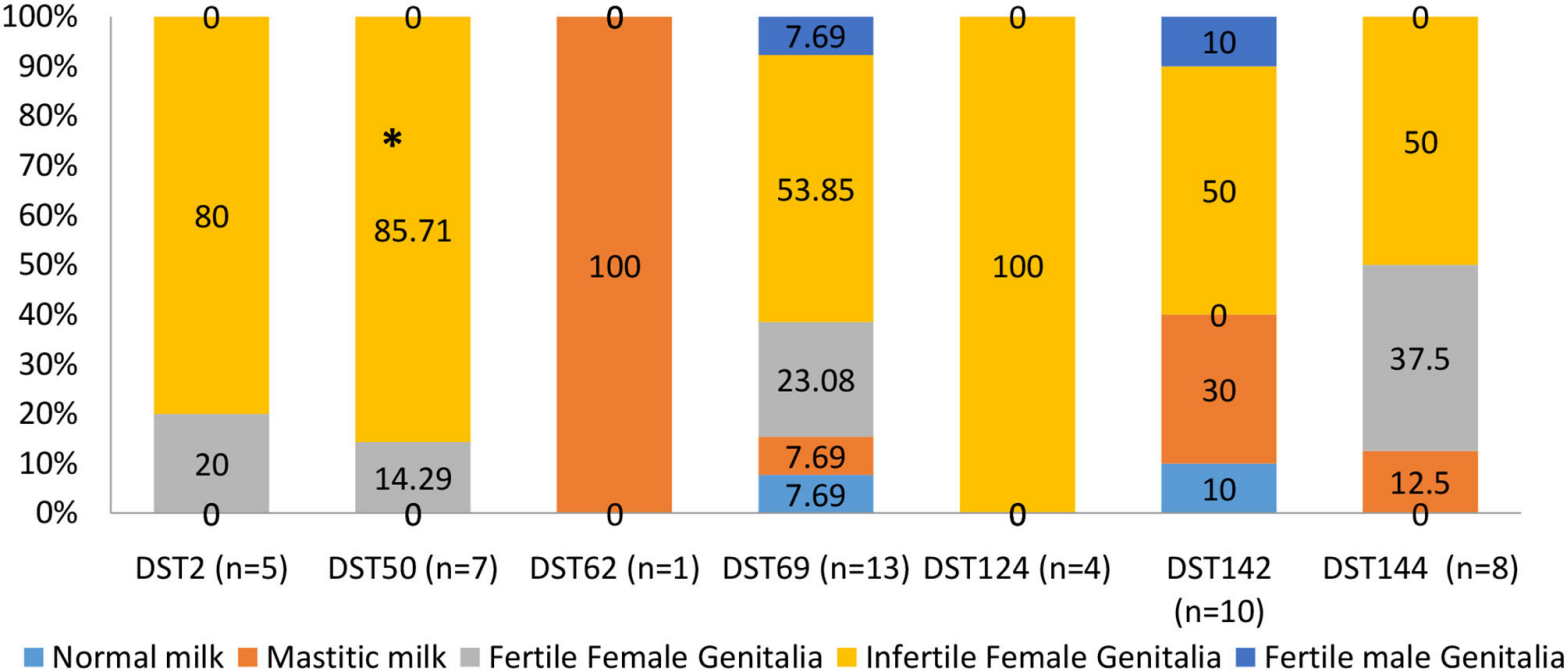
The distribution of different diploid sequence types (DSTs) of *C*. *albicans* isolates in different collected samples of dromedary camels. *Percentage carrying asterisk within the same DSTs differed significantly (*p* ≤ 0.05).

Figure 6 shows the distribution of different DSTs in healthy and diseased camels. DST62 and DST124 were isolated only from diseased camels. DST62 was isolated only from mastitic milk. DST124 was isolated only from infertile female genitalia. The percentage of DST50 and DST 142 was significantly higher in diseased camels (infertile females) than in the apparent healthy ones (fertile females). DST2 and DST50 were isolated only from female genitalia of apparent health and diseased camels.

### The Results of Biofilm Formation Ability, XTT Reduction Assay, CSH Assay and Phospholipase, Proteinase, and Hemolysin Activities

*C. albicans* isolated from diseased camels had significantly higher biofilm formation and phospholipase, proteinase, and hemolysin activities compared with the isolates from apparent healthy camels Table 1.

**Table 1:** Biofilm formation ability, XTT reduction assay and phospholipase, proteinase, and hemolysin activities, and cell surface hydrophobicity assay in the samples collected from milk, female genitalia, and male genitalia of normal and diseased animals (Mean ± Standard Deviation).

	**Normal**	**Diseased**
*N*	12	36
Biofilm formation ability	18.83 ± 6.46	51.23 ± 23.85*
XTT reduction assay	63.35 ± 17*	32.52 ± 19.29
Phospholipase activity	0.95 ± 0.06*	0.86 ± 0.1
Proteinase activity	0.94 ± 0.05*	0.87 ± 0.07
Haemolysin activity	0.91 ± 0.04*	0.78 ± 0.08
Cell surface hydrophobicity assay	5.25 ± 1.90*	10.64 ± 4.15

^*^Means ± standard deviation carrying asterisk within the same row differed significantly (p ≤ 0.05).

**Figure 7:**
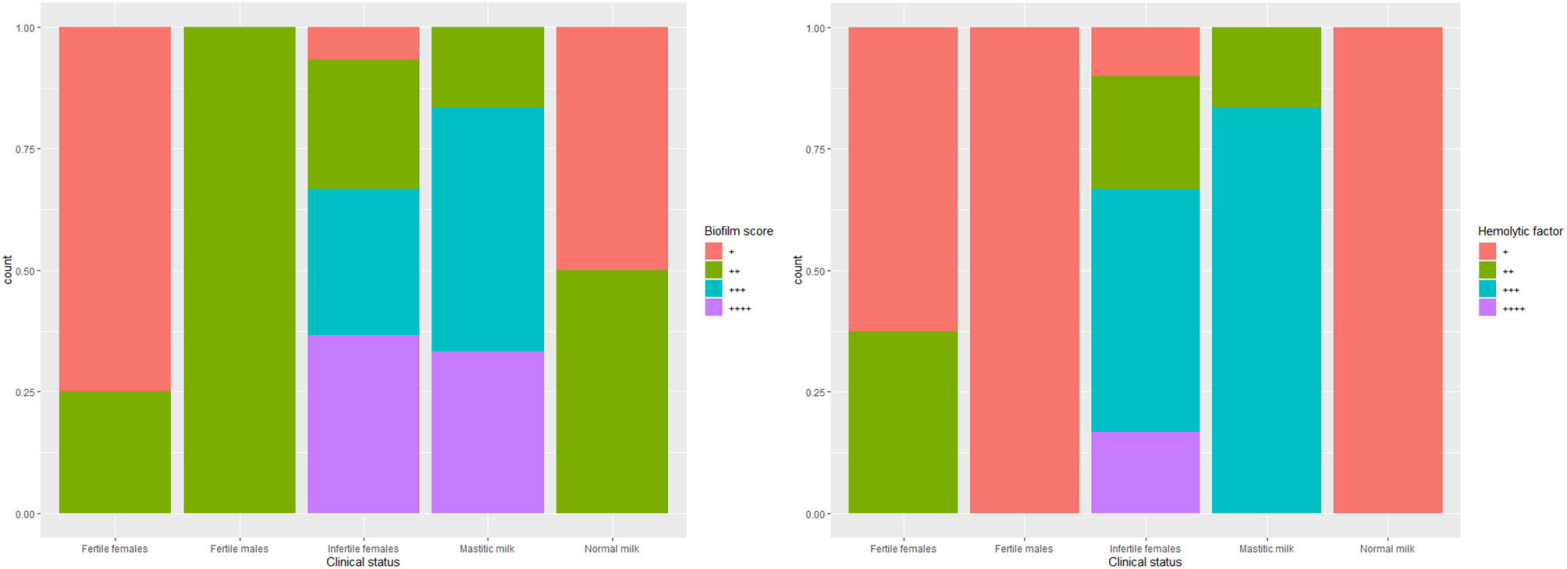
The strength of biofilm formation and hemolysin activities of *C. albicans* isolates from milk and genital tract of dromedary camel.

**Figure 8:**
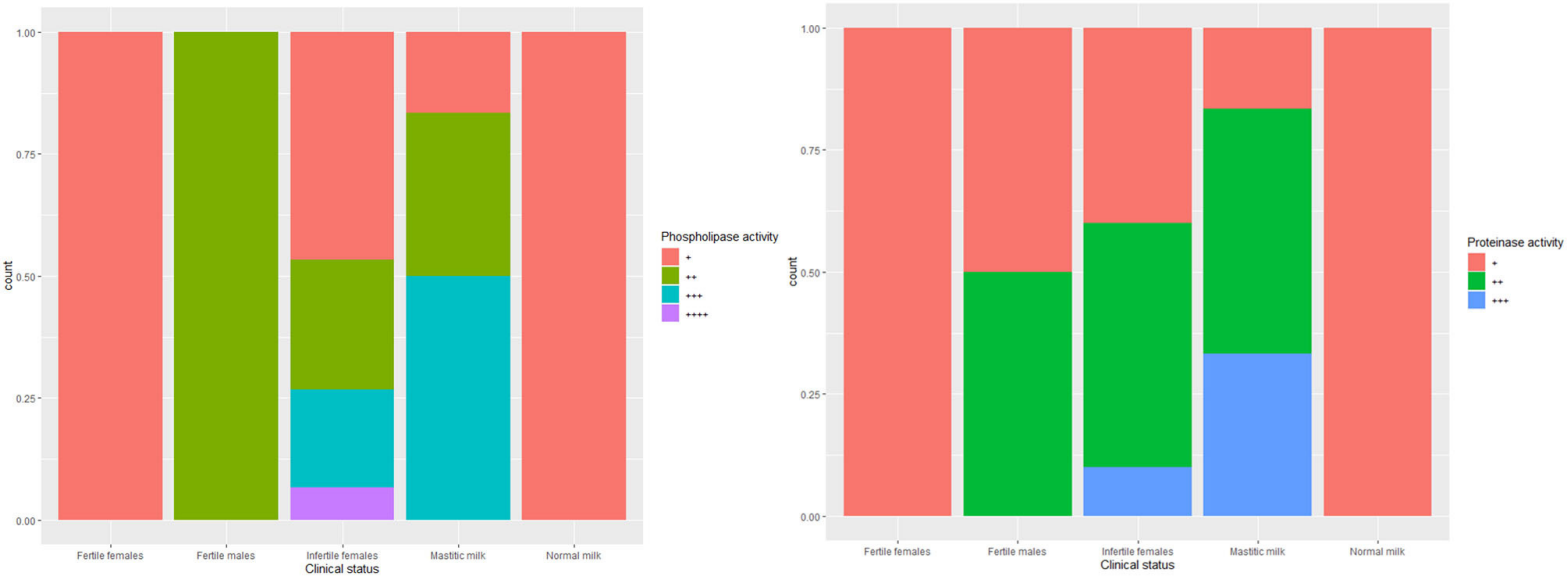
The strength of phospholipase and proteinase activities of *C. albicans* isolates from milk and genital tract of dromedary camel.

The biofilm formation ability was significantly higher for *C. albicans* isolated from mastitic milk than isolates from normal milk and fertile female genitalia. *C. albicans* isolates represented CSH values that ranged from 2.02 to 19.79% with an average of 9.28 ± 4.15. The CSH values were significantly higher in isolates from diseased animals compared with the healthy animal's Table 1. A positive correlation was verified between hydrophobicity and biofilm biomass for *C. albicans* isolates (*r*^2^ 0.885). The phospholipase activity was significantly higher for *C. albicans* isolated from fertile female genitalia than isolates from mastitic milk. The proteinase activity was significantly higher for *C. albicans* isolated from normal milk and fertile female genitalia than isolates from mastitic milk. The hemolysin activity was significantly higher for *C. albicans* isolated from normal milk and fertile female or male genitalia than isolates from mastitic milk and infertile female genitalia Table 2. The strength of biofilm formation, phospholipase, proteinase, and hemolysin activities are illustrated in Figures 7, 8. No significant effect (*p* > 0.05) for the DST on biofilm formation ability, hydrophobicity, XTT reduction assay, and activities of phospholipase, proteinase, and hemolysin was reported in Table 3.

**Table 2:** Biofilm formation ability, XTT reduction assay and phospholipase, proteinase and hemolysin activities, and cell surface hydrophobicity assay in the different animal groups (Mean ± Standard Deviation).

	**Normal milk**	**Mastatic milk**	**Fertile female genitalia**	**Infertile female genitalia**	**Fertile male genitalia**
*N*	2	6	8	30	2
Biofilm formation ability	20.67 ± 8.37^b^	55.70 ± 25.26^a^	16.64 ± 5.93^b^	50.34 ± 23.91^ab^	25.74 ± 1.38^ab^
XTT reduction assay	68.76 ± 24.36^a^	27.55 ± 13.79^b^	65.76 ± 16.75^a^	33.51 ± 20.25^b^	48.33 ± 8.02^ab^
Phospholipase activity	0.96 ± 0.06^ab^	0.82 ± 0.10^b^	0.98 ± 0.03^a^	0.87 ± 0.10^ab^	0.84 ± 0.01^ab^
Proteinase activity	0.96 ± 0.06^a^	0.84 ± 0.09^b^	0.95 ± 0.04^a^	0.87 ± 0.06^ab^	0.88 ± 0.07^ab^
Haemolysin activity	0.93 ± 0.002^a^	0.77 ± 0.03^b^	0.90 ± 0.04^a^	0.78 ± 0.08^b^	0.93 ± 0.01^a^
Cell surface hydrophobicity assay	5.09 ± 1.10^b^	11.45 ± 4.80^a^	5.18 ± 2.33^b^	10.47 ± 4.08^ab^	5.68 ± 0.58^ab^

*^a, b^Means ± Standard Deviation carrying different superscript within the same row differed significantly (p ≤ 0.05)*.

**Table 3:** The relation between the different diploid sequence types (DST) and biofilm formation ability, XTT reduction assay, and activities of phospholipase, proteinase, and hemolysin and cell surface hydrophobicity assay (Mean ± Standard Deviation).

	**DST2**	**DST50**	**DST62**	**DST69**	**DST124**	**DST142**	**DST144**
N*	5	7	1	13	4	10	8
Biofilm formation ability	21.92 ± 9.69	37.26 ± 14.90	85.65	37.95 ± 23.95	60.84 ± 28.79	53.43 ± 25.42	42.89 ± 29.19
XTT reduction assay	61.71 ± 24.84	37.49 ± 18.77	18.05	43.71 ± 25.05	28.92 ± 18.28	30.88 ± 16.52	43.65 ± 25.90
Phospholipase activity	0.90 ± 0.05	0.86 ± 0.11	0.75	0.91 ± 0.09	0.85 ± 0.07	0.87 ± 0.11	0.90 ± 0.13
Proteinase activity	0.92 ± 0.06	0.86 ± 0.07	0.77	0.92 ± 0.07	0.86 ± 0.04	0.87 ± 0.08	0.89 ± 0.05
Haemolysin activity	0.82 ± 0.10	0.78 ± 0.07	0.74	0.82 ± 0.10	0.73 ± 0.04	0.84 ± 0.10	0.83 ± 0.09
Cell surface hydrophobicity assay	6.74 ± 2.89	8.79 ± 3.80	6.14	11.05 ± 5.36	7.91 ± 3.80	9.51 ± 5.08	9.26 ± 4.17

^*^No significant effect was reported (p > 0.05).

### Antifungal Susceptibility Testing

**Figure 9:**
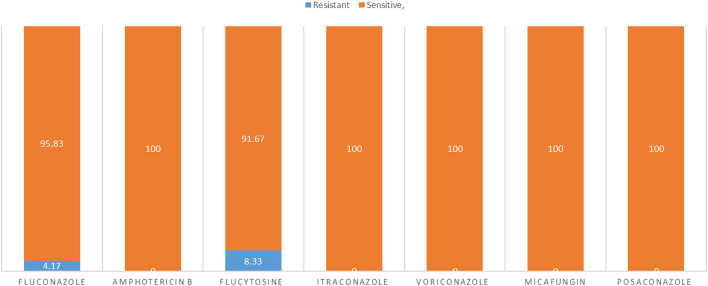
The sensitivity and resistant of *C. albicans* isolates to different types of antifungals.

All isolates were sensitive to amphotericin B, itraconazole posaconazole, micafungin, and voriconazole whereas 2 (4.17%) and 3 (8.33%) of isolates were resistant to fluconazole and flucytosine, respectively (Figure 9).

All resistant isolates belonged to diseased cases. All resistant isolates to fluconazole (*n* = 2) were belonged to infertile female genitalia (DST69 and DST142). While, 75% of resistant isolates to flucytosine (*n* = 3) belonged to infertile female genitalia (DST50, DST124, and DST144) and one belonged to mastitis (DST142). No observed relationship between different DSTs and antifungal susceptibility.

## Discussion

*C. albicans* is the most prevalent fungal pathogen in humans, causing an infection that range from superficial mucosal infections to life-threatening systemic infections (32). *C. albicans* is a common microflora in both human and animal skin and digestive tracts, implying that animals might be vectors of transmission or reservoirs for humans, posing a risk to immunocompromised people (33, 34).

In the present study, 48 *C. albicans* strains were isolated from both apparently healthy and diseases dromedary camels. The frequency of isolation was significantly lower in healthy compared with diseased animals.

Eight (16.67%) isolates were recovered from the genitalia of healthy animals. A wide range of yeast flora including *C. albicans* has been isolated from the genitalia of healthy female camels (35). The role of *C. albicans* as members of female genital flora has been reported in women (36) and different species of animals such as mare (37), cats (38), cow (39), and dog Cleff (9). These results could contribute to an increased understanding of the status of the resident fungal flora in the genitalia of female dromedary camels.

In this work, *C. albicans* was isolated from two normal milk samples. These findings were consistent with previous studies (40–42) that isolated *C. albicans* from raw milk from women and cows. Because of the ubiquitous nature of *C*. albicans, milk samples may be contaminated by the environment or human hands. However, localized and superficial mammary yeast infections have been reported during lactation (43).

Regarding the mastitic milk samples, six *C. albicans* strains were isolated to be consistent with (3, 4, 44); Shokri and Torabi (5) who isolated *C. albicans* from camel mastitis in Ethiopia, Morocco, Egypt, and Iran respectively. Moreover, *C. albicans* was frequently isolated from mastitic milk from cow (45), goat (46), and sheep (47).

In Camelidae, microbial infections of the reproductive system have been identified as a common cause of permanent or temporary infertility and poor reproductive efficiency. Endogenous microorganisms, particularly *Candida* species, can break down natural genital defense mechanisms and cause genital tract infection under predisposing conditions such as breeding, parturition, prolonged antibiotic treatment, and traumatic implantation (48, 49). In this study, the majority of *C. albicans* strains (62.5%) were recovered from the infertile animals. High prevalence of *C. albicans* has been reported in the genital tract of infertile female dromedary camels (10, 50, 51).

The ability of *C. albicans* to colonize the host mucosa and causes the disease is attributed to putative virulence factors such as biofilm formation, hydrophobicity as well as proteinase, phospholipase, and hemolytic activities (52). In this work, *C. albicans* isolates from infertile females and mastitic milk showed a greater biofilm formation and hydrophobicity strength compared with the isolates from healthy animals. Biofilm formation ability is an important attribute of virulence and the majority of *C. albicans*-caused diseases are associated with the formation of biofilms host surfaces (53, 54). Furthermore, biofilms producer *C. albicans* are resistant to killing by neutrophils (55). High CSH is generally considered to be a virulence factor for numerous dimorphic fungal species and a good indicator of adhesion ability (56, 57). A positive correlation was found between biofilm biomass and CSH in our *C. albicans* isolates in agreement with Li et al., Blanco et al., who reported the association between biofilm formation and CSH (58, 59).

Our results showed that 21 (58.3%), 23 (63.9%), and 33 (91.7) of *C. albicans* isolates from diseased animals were positive for phospholipase, proteinase, and hemolytic activities, respectively. These results are relatively in agreement with (60) who found (53%), (83.1%), and (98.8%) of *C. albicans* isolates had phospholipase, proteinase, and hemolytic activities, respectively. Phospholipase destroys the phospholipids in the host cell membrane and subsequently improve invasion and penetration. The proteinase production is considered to enhance the organism's ability to colonize and penetrate host tissue, and to evade the host's immune system by degrading a number of proteins important in host defense. Furthermore, hemolytic capacity allows *Candida* to acquire iron from host tissues for metabolism, growth, and invasion during host infection (26, 52, 61).

The results of antifungal susceptibility testing revealed that 2 (4.17%) and 3 (8.33%) of isolates were resistant to fluconazole and flucytosine, respectively. Fluconazole is fungistatic rather than fungicidal, so treatment provides the opportunity for acquired resistance to develop in the presence of this antifungal (62). Resistance of *C. albicans* to fluconazole has been reported elsewhere (63). Flucytosine-resistant *C. albicans* was detected in Saudi Arabia and Egypt (64). Resistance to flucytosine arises in *C. albicans* as a result of mutations in any of the enzymes involved in the uptake and metabolism of the agent (65).

MLST is a widely used approach to microbial isolate differentiation for epidemiological purposes. Formal MLST scheme have been published for *C. albicans* (17). In this study, seven different DSTs were identified, among them DST69 was the most prevalent diploid sequence type. The identified DSTs were clustered into two clades (Clade1 and clade4). Twenty-six (54.16%) isolates were identified as clade 1; 19 (73.1%) of them were isolated from diseased animals. A significantly greater proportion of isolates associated with superficial infections and commensal carriage in clade 1 *vs*. other clades had been previously reported (66). Clade 1 isolates may be better adapted than others to colonize and invade epithelial surfaces but have no inherent advantage over other types when it comes to traversing epithelia to cause deep-tissue disease (15). In this study, the different DSTs of *C. albicans* showed no significant difference in the virulence factors in consistence with previous studies (67). The three flucytosine-resistant isolates in this study were identified as DST50 and DST69. Clade 1 is particularly rich in flucytosine-resistant isolates (54, 55), and all the flucytosine-resistant isolates from clade 1 that have been studied have the same resistance mechanism: a mutation (R101C) in the FUR1 gene, which encodes uridine phosphoribosyl transferase (68, 69). No data on MLST of *C. albicans* from dromedary camels were available to compare our results. However, the *C. albicans* clade 1 was reported as the most prevalent clade globally, whereas clade 4 is enriched with isolates from the Middle East and Africa (15). It is worth noting that the DST144 (clade4) was reported in Saudi Arabia (67). Furthermore, clade 1 and clade 4 were reported in animals, birds, and wild life (70–72); which may suggest a greater likelihood of *C. albicans* transfer from humans to animals than from animals to humans.

A limitation of this study was the absence of information on the expression of surface and glycoconjugate mannosides. Further investigations on yeast virulence factors in various animal populations are warranted to more precisely evaluate their prognostic value.

## Conclusion

To the best of our knowledge, the present study is the first to report molecular typing by MLST method and phylogenetic analysis of camel *C. albicans* isolated in Saudi Arabia. The frequency of isolation of *C. albicans* was significantly higher in disease camels than in apparently healthy camel. *C. albicans* isolated from infertile female genitalia was significantly higher than that isolated from fertile female genitalia. Multilocus sequence (MLS) analysis identified seven different DSTs including DST2, DST50, DST62, DST69, DST124, DST142, and DST144. The most frequently identified DTS was DST69 which was significantly higher than DST2, DST62, and DST124. The frequency of identification of DST50, DST142, and DST 144 was significantly higher than DST62. DST62 and DST124 were isolated only from diseased camels. DST62 was isolated only from mastitic milk. DST124 was isolated only from infertile female genitalia. The percentage of DST50 and DST 142 was significantly higher in diseased camels (infertile females) than in the apparently healthy ones (fertile females). DST2 and DST50 were isolated only from female genitalia of apparent health and diseased camels. The *C. albicans* isolated from diseased camels had significantly higher biofilm formation and phospholipase, proteinase, and hemolysin activities compared with the isolates from apparent healthy camels. All isolates were sensitive to amphotericin B, itraconazole, micafungin, posaconazole and voriconazole. In conclusion, the present study represents the first molecular typing of *C. albicans* in milk and the genital tract of the dromedary camel. MLST is a useful tool for studying the epidemiology and evolution of *C. albicans*. Biofilm formation is a multifactorial process, involves not only microbial virulence factors but also host immune responses as well as factors related to the composition and shape of the materials used for medical devices. Early identification of *Candida* species and attention to *Candida* virulence factors and their antifungal susceptibility patterns is very important for establishing strategies to control and/or prevent candidiasis by novel therapeutic management. Further studies are needed to evaluate other major virulence factors of *C. albicans* such as a- or b-mannoside expression.

## Data Availability Statement

The datasets presented in this study can be found in online repositories. The names of the repository/repositories and accession number(s) can be found below: https://www.ncbi.nlm.nih.gov/, MT573350 MT594111 MT611969 MT611970 MT611973 MT611971 MT611972 MT611974 MT611975 MT611976 MT604928 MT611977 MT611978 MT611979 MT611980 MT611981 MT611982 MT611984 MT611983 MT611987 MT611988 MT605143 MT611990 MT612078 MT612080 MT612081 MT612079 MT612076 MT612077 MT612082 MT612085 MT612083 MT612087 MT612086 MT612089 MT605404 MT612095 MT612097 MT612096 MT612098 MT605437 MT612099 MT612102 MT612100 MT612101 MT612104 MT612103 MT612106.

## Ethics Statement

The animal study was reviewed and approved by Ministry of Agriculture. Written informed consent was obtained from the owners for the participation of their animals in this study.

## Author Contributions

MF and AS contributed in conceptualization, data curation, formal analysis, investigation, methodology, writing—original draft, and writing—review and editing. AbA, AmA, and FA contributed in investigation and methodology. NA, AhlA, and AhmA contributed in funding acquisition, resources, software, supervision, validation, visualization, roles/writing—original draft, and writing—review and editing. All authors have made substantial contributions to all of the following: (1) the conception and design of the study, or acquisition of data, or analysis and interpretation of data, (2) drafting the article, and (3) final approval of the version to be submitted.

## Conflict of Interest

The authors declare that the research was conducted in the absence of any commercial or financial relationships that could be construed as a potential conflict of interest.

## Publisher's Note

All claims expressed in this article are solely those of the authors and do not necessarily represent those of their affiliated organizations, or those of the publisher, the editors and the reviewers. Any product that may be evaluated in this article, or claim that may be made by its manufacturer, is not guaranteed or endorsed by the publisher.
